# LncRNA MEG3 up-regulates SIRT6 by ubiquitinating EZH2 and alleviates nonalcoholic fatty liver disease

**DOI:** 10.1038/s41420-022-00889-7

**Published:** 2022-03-07

**Authors:** Dongmei Zou, Liang Liu, Yinzhen Zeng, Huanhuan Wang, Dongling Dai, Mingguo Xu

**Affiliations:** 1 Department of Pediatric, Shenzhen Children’s Hospital, China Medical University, 518034 Shenzhen, Guangdong P.R. China; 2 Endoscopy Center, Shenzhen Children’s Hospital, 518034 Shenzhen, Guangdong P.R. China; 3 Department of Pediatric, Longgang District Maternal and Children Health Care Hospital, 518038 Shenzhen, Guangdong P.R. China

**Keywords:** Autoimmune hepatitis, Autoimmunity

## Abstract

Nonalcoholic fatty liver disease (NAFLD) is a global health threat. Here, we presented the significant role of a novel signaling axis comprising long non-coding RNA maternally expressed gene 3 (MEG3), enhancer of zeste homolog 2 (EZH2), and sirtuin 6 (SIRT6) in controlling lipid accumulation, inflammation, and the progression of NAFLD. Mice fed with high-fat diet (HFD) were established as in vitro and in vivo NAFLD models, respectively. Lipid accumulation was measured by oil red O staining and assays for triglycerides or cholesterol. Inflammation was examined by ELISA for pro-inflammatory cytokines. Gene expressions were examined by RT-qPCR or Western blot. Interactions between key signaling molecules were examined by combining expressional analysis, RNA immunoprecipitation, cycloheximide stability assay, co-immunoprecipitation, and chromatin immunoprecipitation. MEG3 level was reduced in FFA-challenged hepatocytes or liver from HFD-fed mice, and the reduction paralleled the severity of NAFLD in clinic. Overexpressing MEG3 suppressed FFA-induced lipid accumulation or inflammation in hepatocytes. By promoting the ubiquitination and degradation of EZH2, MEG3 upregulated SIRT6, an EZH2 target. SIRT6 essentially mediated the protective effects of MEG3 in hepatocytes. Consistently, overexpressing MEG3 alleviated HFD-induced NAFLD in vivo. By controlling the expressions of genes involved in lipid metabolism and inflammation, the MEG3/EZH2/SIRT6 axis significantly suppressed lipid accumulation and inflammation in vitro, and NAFLD development in vivo. Therefore, boosting MEG3 level may benefit the treatment of NAFLD.

## Introduction

Chronic liver diseases from a range of causes other than alcohol consumption are collectively called nonalcoholic fatty liver disease (NAFLD). Involving multiple organ systems and closely associated with other pathological conditions, such as obesity, type 2 diabetes, cardiovascular disease, and chronic kidney diseases [1], NAFLD continuously escalates in its morbidity and mortality, both worldwide and in China [2, 3]. The gold-standard method for diagnosing NAFLD is liver biopsy, which may present as simple steatosis featuring mainly macrovesicular steatosis or as the more severe nonalcoholic steatohepatitis (NASH) featuring additional ballooning, inflammation, and fibrosis [4]. If not properly taken care of, NAFLD frequently progresses into liver fibrosis, liver cirrhosis, and hepatocellular carcinoma. To understand the pathogenic mechanisms underlying NAFLD, many in vitro and in vivo NAFLD models have been developed, such as challenging hepatocytes with free fatty acids (FFA), the key player for NAFLD, or feeding experimental animals with high-fat diet (HFD) [5]. Although these models have significantly contributed to the research on NAFLD, our understanding on NAFLD is still rudimentary and effective therapeutic targets remain to be identified.

Deregulated gene expression, through genetic and/or epigenetic regulations, dictates abnormal lipid metabolism, leading to excessive intracellular lipid accumulation and the development of NAFLD [6, 7]. Consistently, studies have revealed the significance of epigenetic pathways in NAFLD. Among the significant epigenetic regulators, enhancer of zeste homolog 2 (EZH2) is a submit of polycomb repressive complex 2 (PRC2) that catalyzes the trimethylation of histone H3 lysine 27 (H3K27me3) [8]. Studies not only suggest the importance of EZH2 for hepatocyte differentiation and liver homeostasis, but reveal its abnormal activation in NAFLD and its potential as a therapeutic target [9]. However, the mechanisms leading to abnormal activation of EZH2 and mediating its pro-NAFLD effects are mostly unknown. In this study, we aim to identify the upstream regulator of EZH2 and its downstream target that convey its impacts in hepatocytes. Through literature search, we focused on maternally expressed gene 3 (MEG3) and sirtuin 6 (SIRT6).

MEG3 is a long non-coding RNA (lncRNA) critically regulating cell proliferation and suppressing tumor progression [10]. Previous studies have shown that MEG3 may directly interact with EZH2, guiding PRC2 to target genes, and may also promote the ubiquitin-mediated degradation of EZH2 [11–13]. In addition, earlier studies suggest MEG3 was downregulated in NAFLD models [14, 15]; however, no studies have examined the potential crosstalk between MEG3 and EZH2 in NAFLD.

Unlike EZH2 that silences gene expression through histone methylation, SIRT6 activates gene expression by deacetylating histone H3 lysine 9 (H3K9). Studies have suggested the mutual regulation between these two molecules under different conditions: EZH2 may directly silence SIRT6 expression in hepatocytes [16], while SIRT6 may deacetylate EZH2 to regulate FoxC1 expression and ameliorate brain injury [17]. Functionally, SIRT6 has been shown to confer protection against NAFLD [18–20], although it is not clear whether the protection is linked to EZH2.

In the present study, we hypothesize that MEG3, by downregulating EZH2, upregulates SIRT6 and protects against NAFLD. To test this hypothesis, we measured the expression of MEG3 in FFA-stimulated hepatocytes, HFD-induced NAFLD mouse model, examined the interactions between MEG3, EZH2, and SIRT6, and assessed the therapeutic potential of overexpressing MEG3. Through this study, we demonstrate the significance of MEG3/EZH2/SIRT6 axis in NAFLD development and provide new therapeutic targets for the treatment of NAFLD.

## Results

### LncRNA MEG3 was downregulated with the progression NAFLD

**Fig. 1:**
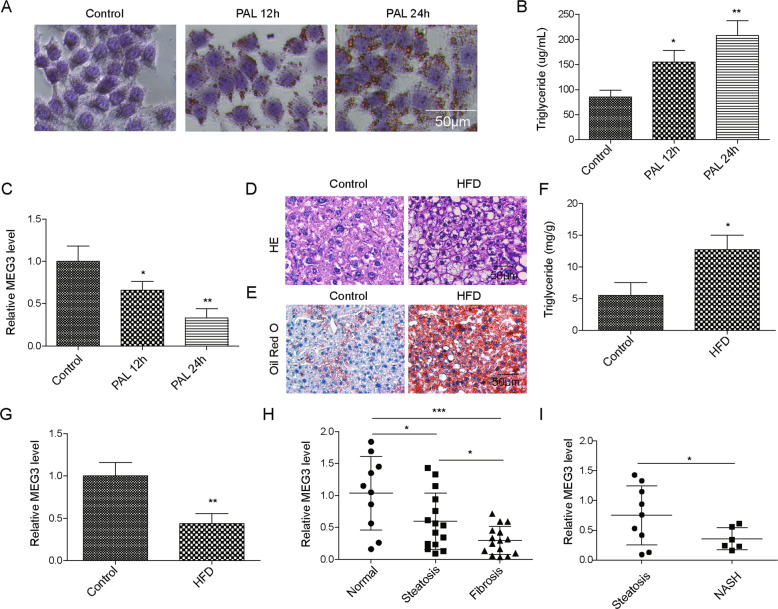
MEG3 was downregulated in NAFLD. **A**–**C** Primary hepatocytes were treated with FFA for 0, 12, and 24 h, respectively. **A** Lipid accumulation was examined by Oil Red O staining. **B** Intracellular triglycerides level was measured by colorimetric assay. **C** MEG3 expression was examined by RT-qPCR. **D**–**G** Mice were fed with HFD for 12 weeks to induce NAFLD. Mice fed with normal diet were used as control. HE staining (**D**) and Oil Red O staining (**E**) of liver tissues from indicated groups. **F** Triglyceride level from the liver tissue was measured. **G** MEG3 expression in liver tissue was examined by RT-qPCR. **H** RT-qPCR was performed to measure MEG3 expression from normal livers (*n* = 10), livers with steatosis (*n* = 15), and those with fibrosis (*n* = 15). **I** RT-qPCR was performed to measure MEG3 expression from patients diagnosed with simple steatosis (*n* = 9) and those from patients with NASH (*n* = 6). *P < 0.05, **P < 0.01.

To understand the potential involvements of MEG3 in NAFLD, we measured its expression in primary hepatocytes challenged with FFA, in liver tissues from HFD-fed mice. Upon challenging hepatocytes with FFA for 12 and 24 h, respectively, we detected increasing lipid accumulation, as shown by Oil Red O staining (Fig. 1A) and the production of intracellular triglyceride (Fig. 1B), suggesting the success of FFA in inducing steatosis. Concomitantly, we observed decreasing expression of MEG3 in these cells (Fig. 1C). In HFD-fed mice, when compared to ND-fed mice, we observed typical NAFLD-related pathological changes in the liver, including hepatocellular ballooning, inflammation, and pericellular fibrosis (Fig. 1D), which was associated with increased formation of lipid droplets (Fig. 1E) and TG production (Fig. 1F). By contrast, MEG3 expression was reduced in liver tissues from HFD-fed mice (Fig. 1G). We observed normal distribution and more robust reduction with more severe NAFLD (Figs. 1H and S1A). MEG3 expression was significantly lower in primary hepatocytes from NASH than in those from simple steatosis (Fig. 1I). Together, these data demonstrate that MEG3 is downregulated following the progression of NAFLD and suggest its potential in inhibiting NAFLD.

### MEG3 protected hepatocytes from FFA-induced NAFLD

**Fig. 2:**
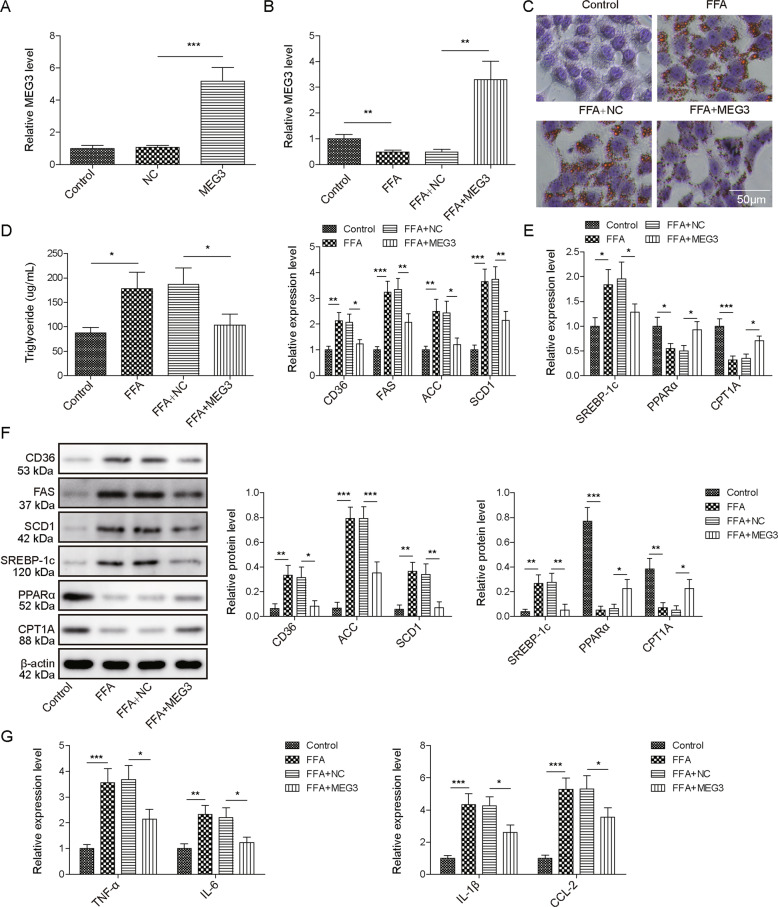
Overexpressing MEG3 protected hepatocytes from FFA challenge. **A** Transfection of hepatocytes with MEG3-expressing vector (MEG3) significantly increased MEG3 level. Non-transfected (control) and those transfected with empty vector (NC) were used as negative controls. **B** MEG3 expression was examined by RT-qPCR in indicated hepatocytes. Oil Red O staining (**C**), triglyceride content (**D**), expressions of indicated genes involved in lipid metabolism on mRNA (**E**) and protein (**F**) levels, and expressions of pro-inflammatory TNF-α, IL-6, IL-1β, and CCL2 (**G**) were examined in indicated hepatocytes. *P < 0.05, **P < 0.01, ***P < 0.001.

To mimic NAFLD condition by treating primary hepatocytes with FFA for 12, 24, and 36 hours, we did perform CCK-8 assay upon treating the hepatocytes for 12, 24, and 36 h (Fig. S1B) and noticed no appreciable hepatocyte degeneration. To assess its functional significance in NAFLD, we overexpressed MEG3 in primary hepatocytes. When compared to vector-transfected hepatocytes (NC), those transfected with MEG3-expressing vector (MEG3) presented significantly boosted expression of MEG3 (Fig. 2A). When challenged with FFA, MEG3 expression was comparably reduced in non-transfected (FFA) and NC (FFA + NC) cells, but remained high in FFA + MEG3 cells (Fig. 2B). Corresponding to different levels of MEG3, we detected abundant formation of lipid droplets (Fig. 2C) and triglyceride production (Fig. 2D) in FFA or FFA + NC cells (*P* < 0.05, when compared to control cells), but not in FFA + MEG3 cells (*P* > 0.05, when compared to control cells). On the molecular level, we noted that FFA robustly altered expressions of genes regulating lipid metabolism, including upregulations of CD36, FAS, ACC1, SCD1, and SREBP-1c, while downregulations of PPARα and CPT1A on the mRNA (Fig. 2E) or the protein (Fig. 2F) levels in FFA and FFA + NC hepatocytes. However, MEG3 overexpression partially or completely reversed the effects of FFA on these genes. Similarly, FFA significantly increased expressions of pro-inflammatory cytokines, including TNF-α, IL-6, IL-1β, and CCL-2 in FFA and FFA + NC cells, but failed to do so in FFA + MEG3 cells (Fig. 2G). These data demonstrate the potency of MEG3 in protecting hepatocytes from FFA-induced lipogenesis and inflammation.

### MEG3 promoted ubiquitin-mediated degradation of EZH2

**Fig. 3:**
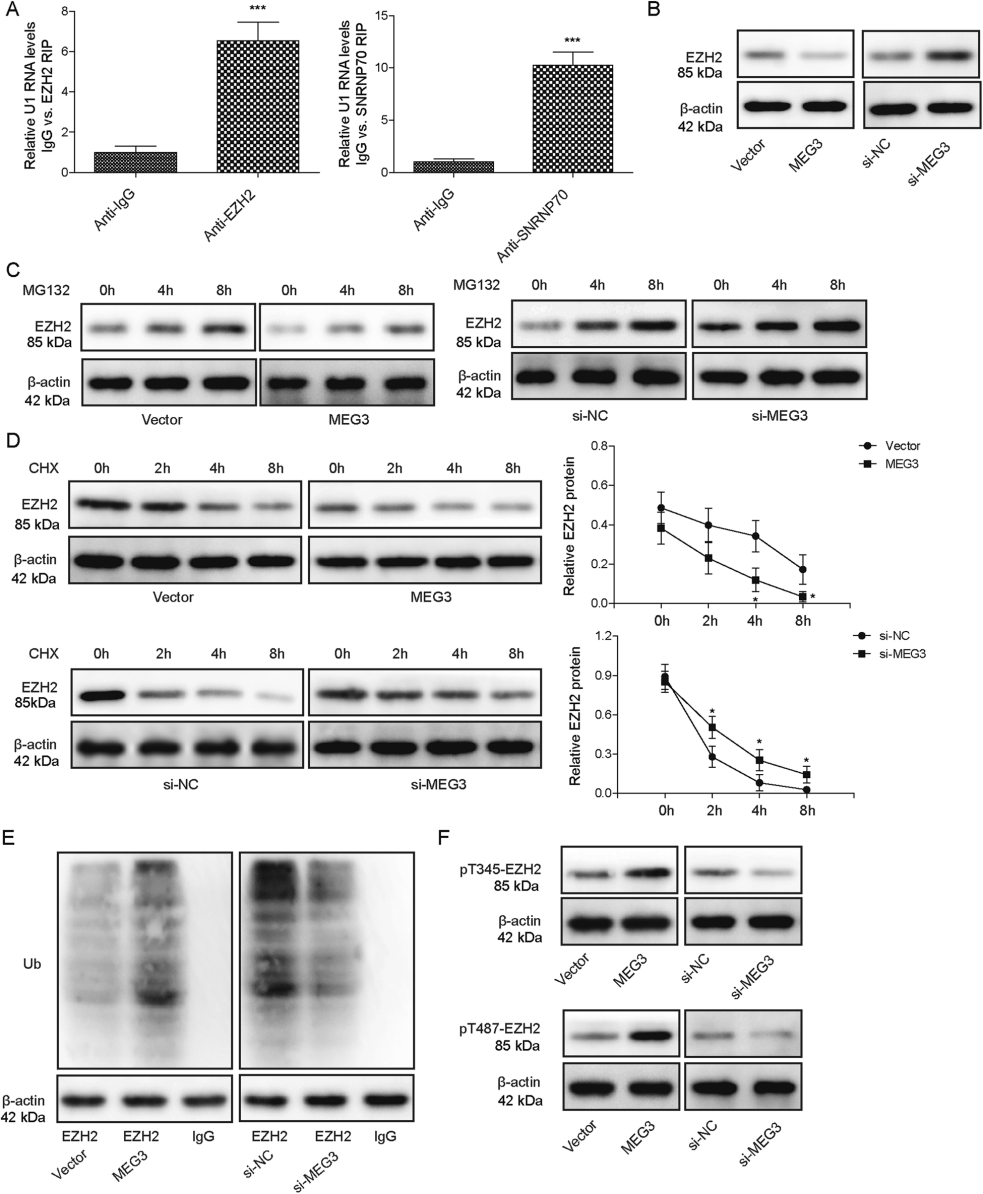
MEG3 targeted EZH2 to ubiquitination-mediated degradation. **A** EZH2 expression was examined by Western blot in MEG3-expressing vs. vector and in si-MEG3-transfected vs. si-NC-transfected hepatocytes. **B** Interaction between MEG3 and EZH2 was examined by RIP assay. IgG was used as the negative control. **C** Indicated hepatocytes were treated with MG132 for 0, 4, and 8 h, respectively. EZH2 level was detected by Western blot. **D** Indicated hepatocytes were treated with cycloheximide for 0, 2, 4, and 8 h, respectively. EZH2 level was detected by Western blot. **E** Ubiquitinated EZH2 was detected by co-immunoprecipitation using either anti-EZH2 or IgG. **F** The levels of phosphorylated EZH2 at T345 and T487 were examined by Western blot in indicated hepatocytes. *P < 0.05, ***P < 0.001.

An earlier study showed that MEG3 downregulated EZH2 by promoting ubiquitin-proteosome degradation of the latter [11]. As a potent factor regulating epigenetic expressions of a variety of gene, activation of EZH2 promotes the development of NAFLD [9]. To understand whether the anti-NAFLD effects of MEG3 are mediated through targeting EZH2, we first measured EZH2 expression in hepatocytes expressing MEG3 or si-MEG3. As shown in Fig. 3A, overexpressing MEG3 significantly reduced EZH2 level, while knocking down MEG3 with si-MEG3 elevated EZH2 expression. RIP assay revealed the direct interaction between MEG3 and EZH2 (Fig. 3B). When treating hepatocytes with MG132, an inhibitor for ubiquitin-proteosome degradation, we detected modest accumulation of EZH2 in NC or si-NC hepatocytes, suggesting ubiquitin-mediated degradation regulates EZH2 protein level in these cells. In contrast, overexpressing MEG3 further promoted, while si-MEG3 suppressed the degradation of EZH2 (Fig. 3C). When blocking de novo protein synthesis with cycloheximide (CHX), we found that overexpressing MEG3 significantly shortened, while si-MEG3 prolonged the half-life of EZH2 (Fig. 3D). Ubiquitination assay revealed increased association between ubiquitin and EZH2 in MEG3-overexpressing cells (when compared to NC cells), while reduced association in si-MEG3 cells (when compared to si-NC cells) (Fig. 3E). Since the hyperphosphorylation of EZH2 at Thr345 and Thr487 precedes the proteasomal degradation of EZH2 [21], we examined the phosphorylation of EZH2 at these two sites in MEG3-overexpressing or MEG3-knockdown hepatocytes. We found that overexpressing MEG3 was sufficient to elevate, while si-MEG3 significantly reduced both pThr345-EZH2 and pThr487-EZH2 levels in hepatocytes (Fig. 3F). Collectively, these data show that MEG3 directly interacts with EZH2, promotes EZH2 phosphorylation at Thr345 and Thr487, and stimulates ubiquitination and proteasome degradation of EZH2.

### SIRT6 mediated the protective effects of MEG3 against FFA-induced lipogenesis and inflammation

**Fig. 4:**
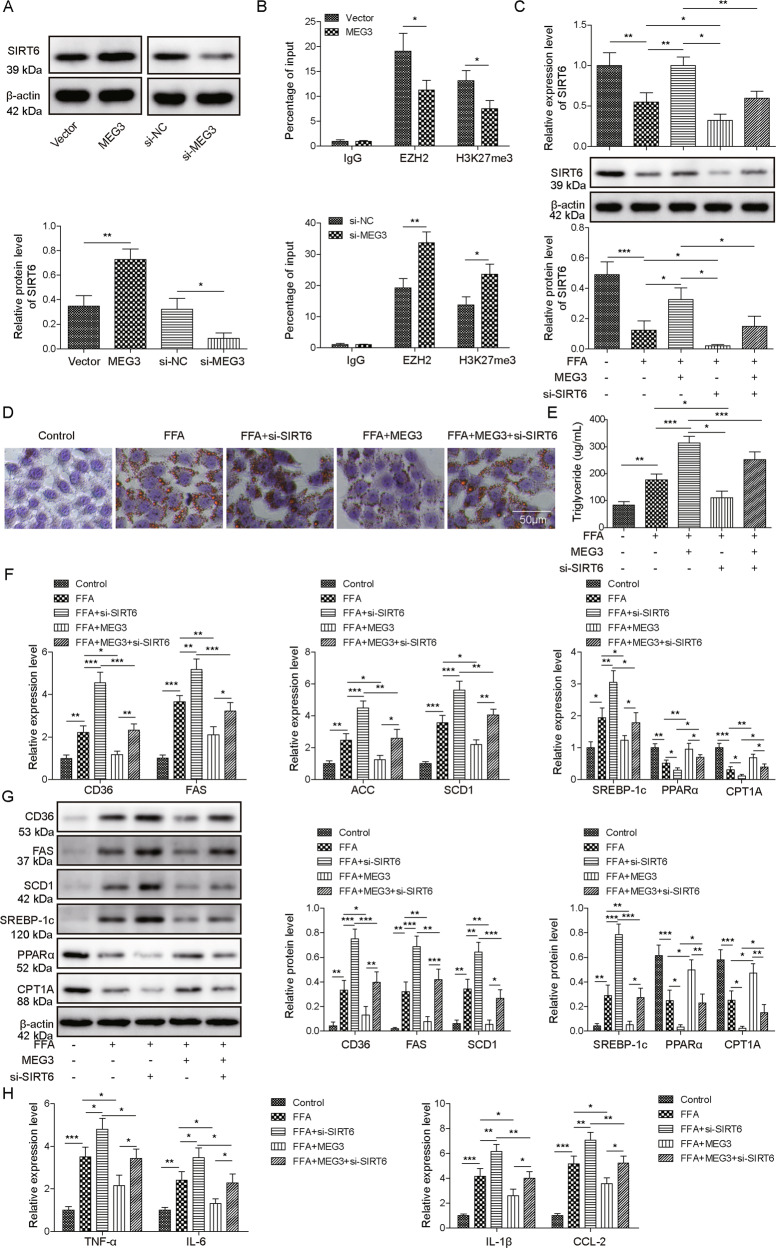
SIRT6 mediated the protection of MEG3 on FFA-challenged hepatocytes. **A** The expression of SIRT6 was measured by Western blot in indicated hepatocytes. **B** Binding of EZH2 and H3K27me3 to the promoter of SIRT6 was detected by ChIP assay. IgG was used as the negative control. **C** SIRT6 expression in indicated hepatocytes was measured by RT-qPCR (upper panel) and Western blot (lower panel). In indicated hepatocytes, Oil Red O staining (**D**), triglyceride assay (**E**), mRNA (**F**) and protein (**G**) of indicated lipogenesis-related genes, and expressions of pro-inflammatory TNF-α, IL-6, IL-1β, and CCL2 (**H**) were performed. *P < 0.05, **P < 0.01*,* ***P < 0.001.

As an essential component in PRC2, EZH2 catalyzes gene silencing through H3K27me3 [8]. Among EZH2 targets, SIRT6 has been shown by earlier studies to inhibit NAFLD [22, 23]. To examine whether SIRT6 may mediate the impacts of MEG3 on NAFLD, we measured its expression in hepatocytes in response to altered levels of MEG3. As shown in Fig. 4A, overexpressing MEG3 upregulated, while knocking down MEG3 reduced SITR6 expression. ChIP assay detected significant binding of EZH2 and H3K27me3 to the promoter of SIRT6 gene in hepatocytes, and the binding was significantly reduced in MEG3-overexpressing cells, but further increased in si-MEG3-expressing cells (Fig. 4B). To assess the functional significance of SIRT6 in NAFLD, we compared the following hepatocytes: vehicle-treated (control), FFA-treated (FFA), MEG3-transfected and FFA-treated (FFA + MEG3), siSIRT6-transfected and FFA-treated (FFA + siSIRT6), and MEG3- and siSIRT6-co-transfected and FFA-treated (FFA + MEG3 + siSIRT6). We found that significant differences existed on SIRT6 mRNA and protein levels between these groups, from the highest to the lowest in the order of control, FFA + MEG3, FFA + MEG3 + siSIRT6, FFA, FFA + siSIRT6 (Fig. 4C). The formation of lipid droplets (Fig. 4D) and intracellular triglyceride production (Fig. 4E), however, were in a reverse order, lowest in control cells, followed by FFA + MEG3, FFA + MEG3 + siSIRT6, FFA, and highest in FFA + siSIRT6 cells, with significant differences in between groups. The same trend was also noted for the expression of CD36, FAS, ACC, SCD1, SREBP-1c (Fig. 4F for mRNA and Fig. 4G for protein), and that of proinflammatory cytokines, including TNF-α, IL-6, IL-1β, and CCL-2 (Fig. 4H), while the reverse trend was noted for PPARα and CPT1A (Fig. 4F, G). Taken together, these data suggest that knocking down SIRT6 is sufficient to aggravate FFA-induced steatosis and to dampen the protection conferred by overexpressing MEG3.

### Overexpressing MEG3 ameliorated HFD-induced NAFLD in vivo

**Fig. 5:**
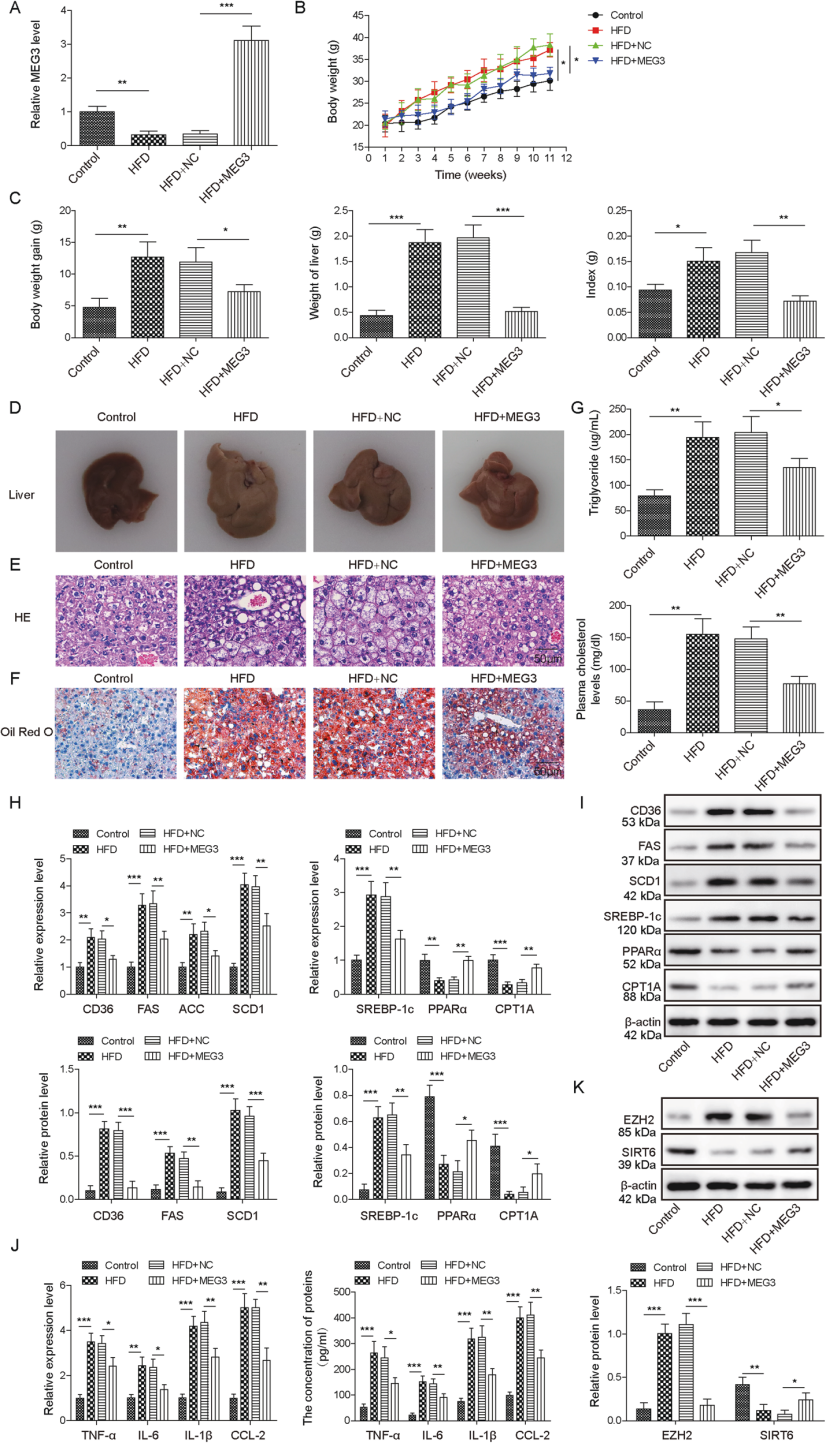
Overexpressing MEG3 alleviated NAFLD in vivo. Empty AAV(NC) or MEG3-expressing AAV was injected into tail vein of mice before these mice were fed with high-fat diet (HFD) for 12 weeks. Mice not injected with AAV (control) were used as the negative control. **A** MEG3 expression in liver tissue was examined by RT-qPCR. **B** The body weight curve for each group. **C** At the time of sacrifice, the body weight, the liver weight, and the liver/body weight index were examined for each group. Representative images of the whole liver (**D**), HE staining (**E**), and Oil Red O staining (**F**) of liver tissues from each group were shown. **G** Serum triglyceride and total cholesterol levels were measured from each group. The mRNA (**H**) and protein (**I**) levels of indicated lipogenesis-related genes, expressions and productions of pro-inflammatory cytokines (**J**), and protein levels of EZH2 and SIRT6 (**K**) were examined in liver tissues from indicated group of mice. *P < 0.05, **P < 0.01, ***P < 0.001.

To investigate the in vivo effects of MEG3 in NAFLD, we injected MEG3-expressing AAV into the tail vein of mice, before feeding mice with HFD (HFD + MEG3). As the negative control, we used either mice fed with ND (control), or those injected with control AAV but fed with HFD (HFD + NC). Upon sacrificing mice after 12-week of HFD, we measured MEG3 expression in liver tissues and found that when compared to control mice, MEG3 was significantly lower (to comparable levels) in HFD and HFD + NC mice, but robustly higher in HFD + MEG3 mice (Fig. 5A), verifying the sustained expression of MEG3 by AAV. In contrast to the changes in MEG3 level, mice in HFD and HFD + NC group achieved the significant gains in body weight, liver weight, and liver/body weight ratio (Fig. 5B, C), which were all significantly inhibited in HFD + MEG3 mice. Correspondingly, liver tissues from HFD and HFD + NC mice presented typical NAFLD morphological (Fig. 5D) and pathological changes (Fig. 5E) with associated increase in the formation of lipid droplets (Fig. 5F), and also in serum triglyceride and cholesterol levels (Fig. 5G). On the molecular level, we detected significant upregulations of CD36, FAS, ACC, SCD1, and SREBP-1c, downregulations of PPARα and CPT1A (Fig. 5H–I), increased expressions and productions of pro-inflammatory TNF-α, IL-6, IL-1β, and CCL-2 (Fig. 5J) in liver tissues from HFD and HFD + NC mice. All these changes were at least partially reversed in HFD + MEG3 mice. Consistent with in vitro findings, we found that EZH2 level was significantly upregulated while SIRT6 downregulated in liver tissues from HFD and HFD + NC mice, but these changes were markedly dampened in liver tissues from HFD + MEG3 mice (Fig. 5K). In collection, these data reveal the benefits of MEG3 in alleviating NAFLD and suggest these benefits are associated with downregulating EZH2, upregulating SIRT6, and suppressing lipogenesis and inflammation.

## Discussion

**Fig. 6:**
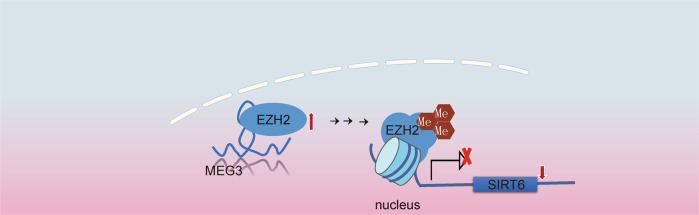
A cartoon image summarizing the major findings of this study.

Dysregulated lipid metabolism leading to lipotoxicity significantly contributes to the development of NAFLD [24]. Combining in vitro and in vivo NAFLD models, as well as clinical NAFLD samples, we presented evidence that MEG3 downregulation is not only a biomarker for NAFLD, but a critical regulator for lipid metabolism and inflammation. The biological activities of MEG3 were mediated through destabilizing EZH2 through ubiquitin-mediated degradation and subsequently upregulating SIRT6 (Fig. 6). When ectopically expressed, MEG3 alleviated HFD-induced NAFLD in vivo. Through this preclinical study, we demonstrate the essential role and the therapeutic value of MEG3/EZH2/SIRT6 axis in NAFLD.

The development of NAFLD involves abnormalities in different biological steps, from FFA uptake and de novo lipogenesis to β-oxidation, triglyceride formation, generation of lipotoxic lipids, to lipid export. This process also engages many cell types including macrophages, fibroblasts, hepatic stem cells, and most importantly, hepatocytes [25]. When using two well-established NAFLD models in this study, FFA-challenged primary hepatocytes and HFD-induced NAFLD mouse, we observed typical NAFLD-related phenotypes, including increased formation of lipid droplets and elevated intracellular or hepatic triglyceride level, demonstrating the success in inducing NAFLD in vitro and in vivo. The downregulation of MEG3 in both models strongly support the importance of MEG3 in NAFLD development and its value as a biomarker for the diagnosis and/or prognosis for NAFLD. Consistently, Huang et al. reported the downregulation of MEG3 in FFA-challenged HepG2 cells as well as in HFD-fed mice [14]. More importantly, we compared MEG3 expression in primary hepatocytes from healthy individuals and patients suffering from earlier steatosis or later-stage fibrosis and revealed the reverse correlation between MEG3 level and the severity of NAFLD, evidencing the clinical relevance of MEG3 in NAFLD.

To understand the biological significance of MEG3, we focused on lipogenesis, the expressional regulation on genes controlling lipid metabolism, as well as inflammation, since overwhelming formation of lipid droplets, triglyceride accumulation, and inflammation are all hallmark features for NAFLD [26]. On the molecular level, these features are closely linked to genes controlling lipid uptake and disposal. Among the genes examined here, CD36 promotes fatty acid uptake [27], FAS, ACC, and SCD1 catalyze de novo lipogenesis [28–30], SREBP-1c transcriptionally activates genes for lipogenesis [31]; PPARα stimulates lipid metabolism and antagonizes inflammation [32], CPT1A mediates fatty acid oxidation [33], and TNF-α, IL-6, IL-1β, and CCL-2 are all pro-inflammatory cytokines/chemokines upregulated in NAFLD [34]. Consistent with their functions, we detected the upregulations of CD36, FAS, ACC, SCD1, SREBP-1c, and all pro-inflammatory cytokines, while the downregulations of PPARα and CPT1A in in vitro and in vivo NAFLD models, and demonstrated the potency of MEG3 in reversing these changes. The therapeutic benefit of MEG3 has also been suggested by Wang et al., where they showed that high-content hydrogen water alleviated NAFLD through upregulating MEG3 [35].

To date, the best characterized mechanism for MEG3, a lncRNA, is by functioning through miRNA/mRNA pathway. For example, MEG3 contributes to hypoxia-induced apoptosis of neurons by sponging miR-181b and upregulating 12/15-LOX [36]. By targeting miR-485/AIM2 axis, MEG3 promotes cerebral ischemia/reperfusion injury [37]. Through miR-34a/Nrf2 pathway, MEG3 protects hepatocytes from ischemia/reperfusion injury [38]. However, acting as a sponge for miRNAs is not the only mechanism for MEG3. MEG3 directly interacts with and elevates Nrf2 protein, but not Nrf2 mRNA in Tenon’s capsule fibroblasts [39]. ChIP followed by high-throughput sequencing revealed the interaction between MEG3 and H3K27me3/EZH2 in PRC2 complex; through the interaction, MEG3 and EZH2 regulate the same set of genes in TGF-β pathway [40]. In this study, we revealed MEG3-mediated targeting of EZH2 to ubiquitination and degradation, which is consistent with a previous study in gallbladder cancer [11].

As the catalytic subunit of PRC2 complex, EZH2 critically regulates liver homeostasis and shapes the development of liver disorders. In NAFLD, however, inconsistent data have been reported on EZH2 level and its functions. Yang et al. reported the upregulation of EZH2 in CCL4-treated liver and its role in promoting liver fibrosis [41]. Martin-Mateos et al. showed that EZH2 essentially mediated TGF-β-mediated liver fibrosis [42]. Lee et al. found that EZH2 was upregulated in a NASH mouse model and EZH2 inhibitors significantly alleviated both liver inflammation and fibrosis [43]. In line with these studies, here we reported the upregulation of EZH2 in NAFLD models, supporting its pathogenic role. In contrast, Vella et al. showed opposite findings that EZH2 was downregulated in HFD-fed rats or FFA-challenged hepatocytes and the downregulation aggravates lipid accumulation and inflammation [44]. Clearly, future studies should carefully examine the status and functions of EZH2 in clinical NAFLD samples.

In addition to revealing the downregulation of MEG3 on EZH2, we also identified SIRT6 as a target for EZH2 and demonstrated its essential role in mediating the protective effects of MEG3 in hepatocytes, that is, inhibiting lipogenesis and inflammation. Cumulative evidence points to the key role of SIRT6 in regulating lipid metabolism and hepatic inflammation. By activating PPARα and subsequently inhibiting SREBP-dependent synthesis of triglyceride and cholesterol, SIRT6 promotes hepatic β-oxidation [45]. Liver-specific silencing of SIRT6 leads to hepatic steatosis and inflammation [20]. Mechanistically, studies have suggested the expressional regulation of SIRT6 on genes involved in lipid metabolism, including those tested in this study, CD36, FAS, ACC, SCD1, SREBP-1c, PPARα, and CPT1A [20, 45, 46].

In summary, this study identifies the MEG3/EZH2/SIRT6 axis and demonstrates its significance in controlling the pathogenesis of NAFLD, specifically, lipogenesis and inflammation. On the cellular level, overexpressing MEG3 was sufficient to alleviate while knocking down SIRT6 promoted FFA-induced lipogenesis and inflammation in hepatocytes. In addition, knocking down SIRT6 dampened the anti-lipogenesis and anti-inflammation benefits of MEG3. This study also provides the first preclinical evidence that boosting MEG3 expression alleviates HFD-induced NAFLD progression. Despite the novel findings, this study leaves many questions to be further addressed. First, is MEG3/EZH2/SIRT6 axis relevant to NAFLD in clinic? Second, what mechanisms lead to the down-regulation of MEG3 following NAFLD development? Third, is there any crosstalk between this axis and other MEG3-regulated signaling pathways? Understanding these questions will benefit the design of specific and potent anti-NAFLD therapies.

## Materials and methods

Procedures involving experimental animals were reviewed and approved by the Ethics Committee of Shenzhen TopBiotech Co., Ltd.

### In vivo NAFLD model

All animal procedures were approved by the Animal Ethics Committee of Shenzhen TopBiotech Co., Ltd. HFD-induced NAFLD mouse model was established as described before [47]. Briefly, six-week-old male C57/BL6 mice were purchased Shanghai SLAC Laboratory Animal Co. (Shanghai, China) and acclimatized to the housing environment for one week. To overexpress MEG3 in vivo, adeno-associated virus (AAV) expressing MEG3 or control AAV (Genomeditech) was injected into the tail vein at 1 × 10^11^ vector genomes/mouse on Day 0. Then mice were randomly and equally divided into groups and were fed with either normal diet (ND; 10% kcal% fat; D12450B) or HFD (60% kcal% fat; D12492; Research Diets, New Brunswick, NJ, USA). Access to water was not limited. All mice were monitored for their body weight on a weekly basis. After 12 weeks, mice were sacrificed, whole blood was collected from the heart, and liver tissues were dissected, weighted, and further analyzed.

### Real-time quantitative PCR (RT-qPCR)

Total RNA was extracted from mouse tissues using Total RNA Extraction Kit (Cat. No. R1200; Solarbio, Beijing, China). Reverse transcription was performed using Universal RT-qPCR kit (Cat. No. RP1100; Solarbio) and RT-qPCR using 2× SYBR Green PCR Mastermix (Cat. No. SR1110; Solarbio). Primers used for RT-qPCR analysis (Table 1), including those for mouse MEG3, EZH2, SIRT6, CD36, fatty acid synthase (FAS), acetyl-CoA carboxylase 1 (ACC1), stearoyl-CoA desaturase-1 (SCD1), sterol regulatory element binding protein-1c (SREBP-1c), peroxisome proliferator-activated receptor alpha (PPARα), carnitine palmitoyltransferase 1A (CPT1A), tumor necrosis factor α (TNF-α), interleukin 6 (IL-6), IL-1β, chemokine ligand 2 (CCL-2), and GAPDH (internal control for mRNAs) were synthesized by GeneChem (Shanghai, China). Relative gene expression was calculated using 2^−ΔΔCt^ method.

**Table 1:** Sequences for primers used for RT-qPCR assay.

Gene	Species	Forward primer (5′-3′)	Reverse primer (5′-3′)
MEG3	Mouse	CACAGAAGACGAAGAGCTGGA	GGTAGAGGTGCACAGCAGGT
		TTTTGTGCCCAAGGCTCCTGGA	AGGGACTCAAGGAGCCAGGTTA
EZH2	Mouse	CATACGCTCTTCTGTCGACGATG	ACACTGTGGTCCACAAGGCTTG
		GACCTCTGTCTTACTTGTGGAGC	CGTCAGATGGTGCCAGCAATAG
SIRT6	Mouse	CAGTACGTCAGAGACACGGTTG	GTCCAGAATGGTGTCTCTCAGC
		TGGCAGTCTTCCAGTGTGGTGT	CGCTCTCAAAGGTGGTGTCGAA
CD36	Mouse	GGACATTGAGATTCTTTTCCTCTG	GCAAAGGCATTGGCTGGAAGAAC
FAS	Mouse	CTGCGATTCTCCTGGCTGTGAA	CAACAACCATAGGCGATTTCTGG
ACC1	Mouse	GTTCTGTTGGACAACGCCTTCAC	GGAGTCACAGAAGCAGCCCATT
SCD1	Mouse	GCAAGCTCTACACCTGCCTCTT	CGTGCCTTGTAAGTTCTGTGGC
SREBP-1c	Mouse	CGACTACATCCGCTTCTTGCAG	CCTCCATAGACACATCTGTGCC
PPARα	Mouse	ACCACTACGGAGTTCACGCATG	GAATCTTGCAGCTCCGATCACAC
CPT1A	Mouse	GGCATAAACGCAGAGCATTCCTG	CAGTGTCCATCCTCTGAGTAGC
TNF-α	Mouse	GGTGCCTATGTCTCAGCCTCTT	GCCATAGAACTGATGAGAGGGAG
IL-6	Mouse	TACCACTTCACAAGTCGGAGGC	CTGCAAGTGCATCATCGTTGTTC
IL-1β	Mouse	TGGACCTTCCAGGATGAGGACA	GTTCATCTCGGAGCCTGTAGTG
CCL-2	Mouse	GCTACAAGAGGATCACCAGCAG	GTCTGGACCCATTCCTTCTTGG
GAPDH	Mouse	CATCACTGCCACCCAGAAGACTG	ATGCCAGTGAGCTTCCCGTTCAG

### Oil red O staining and measurement of triglyceride and cholesterol

For oil red O staining, mouse tissues (with 10% formalin) were incubated with 0.5% oil red O in isopropanol (Sigma) for 20 min, and then counterstained using hematoxylin (Abcam, Cambridge, MA, USA) for 1 min. The triglyceride or cholesterol level from hepatocytes or mouse serum was measured using Triglyceride Assay kit (ab65336) or Cholesterol Assay kit (ab65359; Abcam) following the manufacturer’s instructions.

### Histological analysis

Liver tissues were fixed in 10% formalin for 24 h and embedded in paraffin before 4-μm tissue sections were prepared. Hematoxylin and eosin staining was performed using HE Staining kit (Cat. No. ab245880; Abcam) following the instructions from the manufacturer.

### ELISA

The content of TNF-α, IL-6, IL-1β, and CCL-2 in mouse liver was analyzed according to the protocol from ELISA kit for the corresponding mouse genes (Cat. No. E0117Mo for TNF-α; E0049Mo for IL-6; E0192Mo for IL-1β; E1707Mo for CCL-2; Bioassay Technology Lab, Shanghai, China).

### RNA immunoprecipitation (RIP)

The association of MEG3 with EZH2 was examined following the instructions from RIP assay kit (Cat. No. 17-700; Sigma). In brief, hepatocyte lysates were incubated with either anti-EZH2 antibody (ab195409) or normal IgG (ab109489; Abcam) followed by protein A magnetic beads. The co‐precipitated RNA was extracted with a RNeasy MinElute Cleanup Kit (Cat. No. 74204; Qiagen) and then detected by RT-qPCR.

### Ubiquitination assay

To examine EZH2 ubiquitination, hepatocytes was lysed in a buffer containing 20 mM Tris HCl pH 8, 137 mM NaCl, 1% Nonidet P-40, 2 mM EDTA, and 1× protease/phosphatase inhibitor cocktail (Cell Signaling, Danvers, MA, USA). After incubating with 1 μg anti-EZH2 antibody (ab195409) or rabbit IgG (both from Abcam) on at 4 °C for 1 h followed by protein A sepharose (Abcam) at 4 °C overnight, the Sepharose beads were washed, boiled in 5× sample loading buffer, and examined using Western blot.

### Chromatin immunoprecipitation (ChIP)

ChIP assay was performed according to the instructions from One-Step ChIP kit (Cat. No. ab117138; Abcam). In brief, target cells were crosslinked with 1% formaldehyde before lysed to generate nuclei. Then chromatin was sonicated to generate DNA/protein fragments of 150–900 base pairs (bps) in length. Upon incubating the digested chromatin with anti-EZH2 (ab195409), anti-H3K27me3 (ab6002), or IgG (all from Abcam) at 4 °C overnight, the immune complexes were immunoprecipitated with ChIP-grade protein G magnetic beads. DNA pulled down by the antibody was examined by RT-qPCR.

### Western blot

Cell lysates were prepared from mouse liver tissues using RIPA buffer (Beyotime) supplemented with protease and phosphatase inhibitors. Equal amounts of total proteins were separated on SDS-PAGE gel and transferred onto polyvinylidene fluoride membranes. After blocking the membrane in Tris-buffered saline with Tween (TBST) buffer containing 5% non-fat milk powder for 1 h, the membrane was incubated at 4 °C overnight with primary antibodies for the following proteins (all from Abcam or stated otherwise): CD36 (1:1000, ab133625), FAS (1:1000, ab216991), ACC1 (1:1000, ab45174), SCD1 (1:1000, ab236868), SREBP-1c (1:1000, ab28481), PPARα (1:1000, ab126285), CPT1A (1:1000, ab234111), EZH2 (1:1000, ab195409), p-EZH2 (T345) (1:1000, PA5-114574; Thermo Fisher), p-EZH2 (T487) (1:1000, PA5-105660, Thermo Fisher), SIRT6 (1:1000, ab191385), ubiquitin (1:1000, ab140601), or β-actin (1:3000, ab8226). After three washes with TBST, the membrane was incubated with horseradish peroxidase-labeled secondary antibodies and signals were developed with enhanced chemiluminescence substrate and imaged using LAS400 imaging system (Fujifilm, Tokyo, Japan).

### Statistical analysis

All data were analyzed with SPSS software (version 22.0; IBM, Armonk, NY, USA). Quantitative data were presented as mean ± SD, tested for normality, and compared using Student’s *t*-test between two groups and ANOVA followed by Tukey’s post hoc test among multiple groups. Statistical significance was defined as *P* < 0.05.

## Supplementary information

supplementary information

## Data Availability

All data generated or analysed during this study are included in this published article
