# Supplementary material for: LncRNA MEG3 up-regulates SIRT6 by ubiquitinating EZH2 and alleviates nonalcoholic fatty liver disease
Source: Cell Death Discov. 2022 Mar 7;8:103. doi: 10.1038/s41420-022-00889-7 (PMC8901640; doi:10.1038/s41420-022-00889-7)
Supplement: Supplementary file 1 — supplementary information [file 41420_2022_889_MOESM1_ESM.pdf]

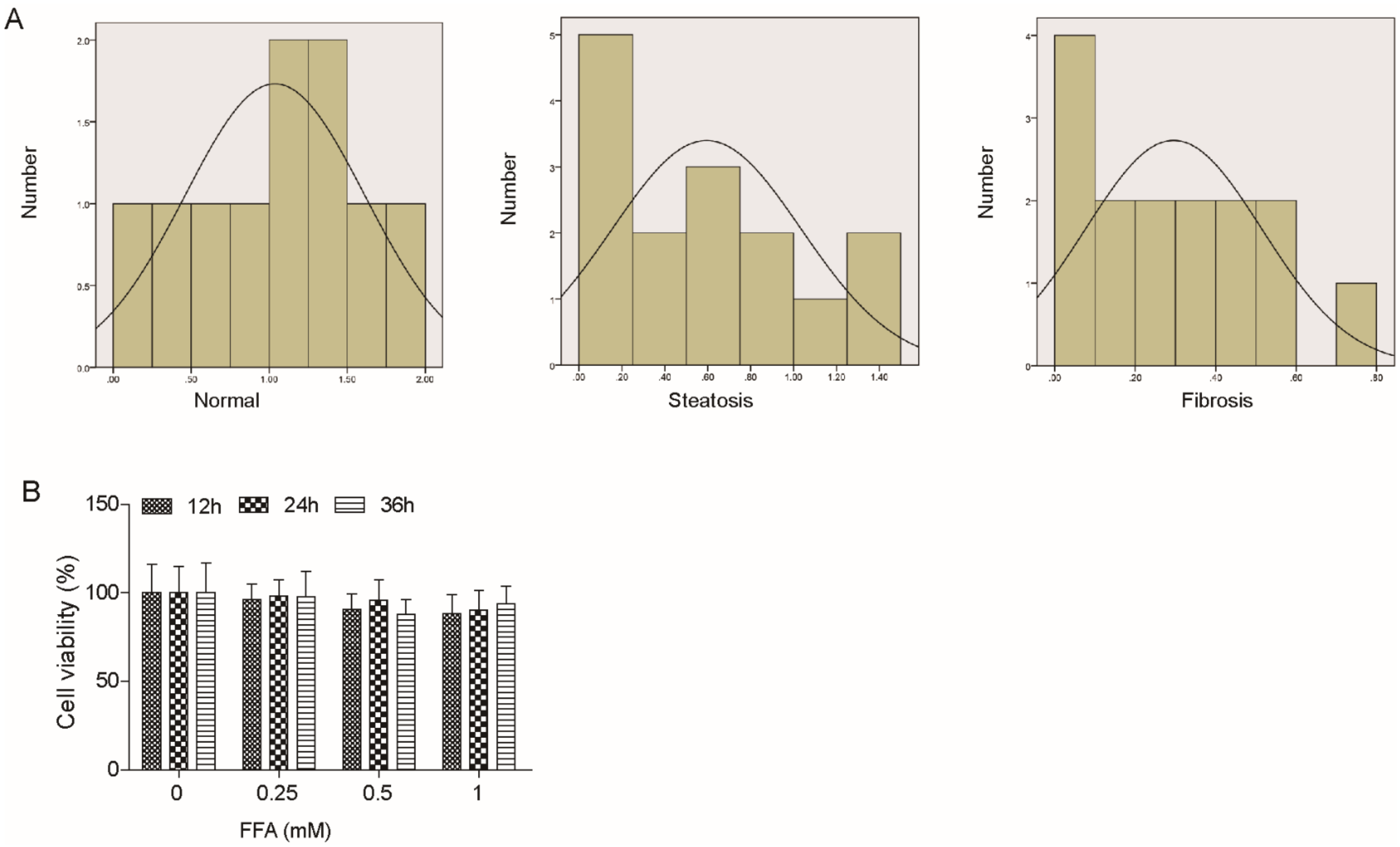

Figure S1. The normal distribution and cell viability were performed.

(A) The normal distribution of MEG3 expression was performed in patients' data from normal livers (n=10), livers with steatosis (n=15), and those with fibrosis (n=15). (B) CCK-8 assay were performed to valuate cell viability upon treating the hepatocytes for 12, 24 and 36 h.
